# Anti-tumor and anti-metastatic effects of RRx-001 on hepatocellular carcinoma: mechanisms of action and therapeutic potential

**DOI:** 10.3389/fphar.2024.1469825

**Published:** 2024-11-27

**Authors:** Guohong Yan, Shuqi Zhao, Meifeng Chen, Shutian Mo, Hailian Huang, Yuan Liao, Ziyan Lu, Jiaming Liang, Shuxin Wei, Chuangye Han, Xinping Ye

**Affiliations:** ^1^ Department of Hepatobiliary Surgery, The First Affiliated Hospital of Guangxi Medical University, Nanning, China; ^2^ School of Basic Medical Sciences, Guangxi Medical University Nanning, Nanning, China; ^3^ Department of Hepatobiliary Surgery, Nanyang Central Hospital, Nanyang, China

**Keywords:** hepatocellular carcinoma (HCC), RRx-001, TP53, cd47, immunology

## Abstract

**Background:**

1-Bromoacetyl-3,3-dinitroazetidine (RRx-001) has potent antitumor effects, indicating its promising therapeutic potential against various cancers. This research investigates RRx-001 activity against hepatocellular carcinoma (HCC) and elucidates its underlying mechanisms.

**Methods:**

Huh7, Hepa1-6, and MHCC97H cells were cultured and treated with varying RRx-001 concentrations for 24, 48, and 72 h. Cell viability was assessed using cell counting kit-8. The cells were divided into control and RRx-001 treatment groups at 0.5 × IC_50_, 1.0 × IC_50_, and 2.0 × IC_50_ concentrations for each cell line. Migration and invasion were evaluated using scratch and Transwell assays, and apoptosis was examined by apoptosis assays. RNA sequencing was performed on the Huh7 cells treated with RRx-001 for 24 h to identify differential gene expression. CD47 and TP53 protein levels were measured by Western blot. A xenograft mouse model was utilized to evaluate the effect of RRx-001 on HCC.

**Results:**

RRx-001 inhibits HCC cell viability, migration, and invasion while inducing apoptosis, These effects are potentially mediated by the downregulation of CD47 and the upregulation of TP53, both of which modulate key signaling pathways. *In vivo* experiments demonstrated that RRx-001 effectively inhibits tumor growth.

**Conclusion:**

RRx-001 reduces the viability of HCC cells and induces apoptosis. This effect may be due to the downregulation of CD47 expression and the alteration of the TP53 protein regulatory pathway.

## 1 Introduction

Primary liver cancer is one of the most prevalent malignancies globally, with HCC being the most common type. Early diagnosis of HCC remains challenging, resulting in many patients being diagnosed at advanced stages (Sun et al., 2020; Sung et al., 2021). Systemic therapy, including targeted therapies and immunotherapies, is the main treatment for advanced-stage HCC. The most commonly utilized drugs fall into two categories: targeted therapies and immunotherapies. Targeted therapies primarily include tyrosine kinase inhibitors, such as sorafenib and lenvatinib, and antiangiogenic agents. Meanwhile, PD-1 and PD-L1 monoclonal antibodies are the most frequently employed in immunotherapy (Cheng et al., 2020; Yi et al., 2022; Bray et al., 2024). Despite the advancements in both therapies, a significant proportion of patients experience adverse reactions and develop drug resistance, diminishing the efficacy of treatment (Zheng et al., 2017; Raghavendra et al., 2018). Hence, exploring effective drugs and treatment strategies is urgently needed to improve clinical outcomes for patients with HCC.

The significance of studying RRx-001 lies in its multifaceted mechanisms of action and its broad therapeutic potential. In preclinical studies, it has demonstrated neuroprotective effects in neurodegenerative disorders such as Parkinson’s disease, Alzheimer’s disease, multiple sclerosis, and amyotrophic lateral sclerosis. Furthermore, due to its radiosensitizing properties, RRx-001 is considered a promising therapeutic option, particularly effective in mitigating the effects of high-dose radiation exposure from radiological or nuclear incidents. RRx-001 is the world’s first small molecule drug targeting the CD47 signaling pathway and currently the only CD47-targeted oncology drug to have reached Phase III clinical trials. It has shown remarkable anti-cancer efficacy across a variety of tumors, including colorectal cancer, lung cancer, osteosarcoma, ovarian cancer, and glioma (Jurgensen et al., 2021; Reid et al., 2022; Morgensztern et al., 2019; Das et al., 2016; Cottrill et al., 2018; Oronsky et al., 2021; Fang et al., 2022).The anti-cancer mechanisms of RRx-001 are varied and include inhibiting the CD47-SIRPαpathway, which repolarizes tumor-associated macrophages (TAM) from an anti-inflammatory M2 phenotype to a pro-inflammatory M1 phenotype. Additionally, it reactivates tumor-suppressor genes via epigenetic modulation to overcome chemotherapy resistance, normalizes tumor vasculature to improve drug permeability, and induces tumor cell necrosis through the generation of reactive oxygen and nitrogen species (RONS) (Fens et al., 2016; Scicinski et al., 2015a). Clinical trials have shown that RRx-001 possesses a favorable safety profile and efficacy in treating solid tumors. For instance, it has progressed to Phase III clinical trials in combination with platinum-based chemotherapeutic agents for third-line treatment of small cell lung cancer (SCLC) (Oronsky et al., 2019a). Furthermore, the KEVLARx project is conducting a Phase IIb radioprotection trial for head and neck cancer. These findings suggest that RRx-001 holds great promise as a novel therapeutic approach, with the potential to significantly improve patient survival outcomes and expand treatment options.

## 2 Materials and methods

### 2.1 Cell culture

Human HCC cell lines Huh7 and MHCC97H and mouse HCC cell line Hepa1-6 were obtained from the Kunming Cell Bank and Shanghai Cell Bank of the Chinese Academy of Sciences, respectively. DMEM (C04001-500) and fetal bovine serum (C3113-0500) were purchased from Shanghai Xiaopeng Biotechnology Co., Ltd., and 1% penicillin-streptomycin (03-031-5B) was procured from BI Company, Israel. The cell culture conditions were set at 37°C with CO_2_ concentration of 5%.

### 2.2 CCK8 assay for Huh7, Hepa1-6, and MHCC97H cell viability

Cells in logarithmic growth phase (Huh7, Hepa1-6, and MHCC97H) were adjusted to a density of 5 × 10^4^ cells/mL and seeded in 96-well plates with 100 μL per well. The cells were then incubated overnight at 37°C in a carbon monoxide incubator. Experimental groups with different RRx-001 concentrations and control groups were set up, and the cells were incubated for 24, 48, and 72 h. Each well was added with 10 μL of CCK8 (BS350B, BS) solution, followed by a 2-h incubation. Absorbance was measured at 450 nm using a microplate reader. Cell viability (%) was calculated as [(OD of experimental group − OD of control group)/(OD of control group − OD of blank group)] × 100% to determine the half-maximal inhibitory concentration (IC_50_) of RRx-001 against Huh7, Hepa1-6, and MHCC97H cells.

### 2.3 Scratch assay

Scratch assay was conducted to evaluate cell migration ability. Cells (Huh7, Hepa1-6, and MHCC97H) in logarithmic growth phase were seeded in six-well plates at a density of 2 × 10^5^ cells/mL and incubated at 37°C with 5% CO_2_ for 24 h. Once the cells reached confluence, scratches were made. The cells in different groups were treated with various RRx-001 concentrations in 2% FBS culture medium for 24 h. The cells were then observed under an inverted optical microscope, images were captured, and the cell migration area was calculated with ImageJ software. Migration assay was performed to assess cell migration capability. Migration rate (%) = (Scratch width at 0 h - Scratch width at treatment time)/Scratch width at 0 h × 100 %

### 2.4 Transwell analysis

For migration and invasion assays, Transwell chambers (8 μm pore size, BD) were coated with Matrigel (354248, Corning) and incubated at 37°C for 1 h to form a gel. For migration assays, the Matrigel coating was not required. Huh7, Hepa1-6, and MHCC97H cells were treated with different RRx-001 concentrations for 24 h. The cells were collected and resuspended in serum-free medium, and 200 μL of cell suspension (8 × 10^4^ cells/mL) was added to the upper chamber of the Transwell plate. Meanwhile, the lower chamber contained 700 μL of medium containing 10% FBS. After incubation for 24 h, the cells were fixed with 4% paraformaldehyde, stained with crystal violet (0.1%) for 45 min at room temperature, After washing and drying, the number of penetrated cells was recorded by photography under an inverted microscope, and the number of penetrated cells was used as a judgment index to evaluate the migration and invasion ability of cells.

### 2.5 Flow cytometric analysis of apoptosis

Huh7, Hepa1-6, and MHCC97H cells were dissociated, adjusted to a density of 2 × 10^5^ cells, seeded in six-well plates, and incubated at 37°C with 5% CO_2_ for 24 h. The cells were treated with varying RRx-001 concentrations for 24 h. Cell apoptosis was detected using the Annexin V-FITC apoptosis detection kit (C1062M, Beyotime), and the distribution of apoptotic cells was analyzed using flow cytometry.

### 2.6 RNA-seq analysis

#### 2.6.1 Library construction and sequencing

Logarithmic-phase Huh7 cells were adjusted to a cell density of 1 × 10^6^ cells/mL and seeded into 6-well plates with 2 mL of cell suspension per well. Cells were cultured in DMEM high-glucose medium for 24 h. Experimental groups were set at IC_50_ concentration, and a blank control group was included. After 24 h of treatment, cells were collected. Experiments were repeated three times. RNA extraction, PCR amplification, library construction, and sequencing were conducted by Shanghai Sangon Biotech Co., Ltd.

#### 2.6.2 Differential gene expression analysis

Differential expression analysis of component genes was performed with edgeR software. Differential genes were screened using FDR and log2FC, with selection criteria set at *P* < 0.05 and |log2FC| > 1.

#### 2.6.3 Gene ontology (GO)/pathway enrichment analysis of differential genes

The GO database was utilized to perform GO biological function analysis on differential genes, and a GO biological process distribution map was constructed. Kyoto Encyclopedia of Genes and Genomes (KEGG) was used for signal pathway enrichment, annotation, and analysis of differential genes. For gene function and signal pathway enrichment analyses, FDR <0.01 was set as the threshold, with *P*-value adjustment for validation.

### 2.7 Western blot analysis

Cells (Huh7, Hepa1-6, and MHCC97H) were grouped and treated for 48 h. Protein was extracted by adding RIPA rapid cell lysis buffer (P0013B, Beyotime), PSMF (ST507-10 mL, Beyotime), and protein phosphatase inhibitors (P1005, Beyotime). The protein concentration was determined using the BCA method, and the samples were mixed with protein loading buffer at a ratio of 4:1. The proteins were separated by 10% SDS-PAGE, transferred to a PVDF membrane, blocked with 5% skim milk for 1 h, and then incubated overnight at 4°C with β-actin (66009-1-Ig), CD47 (20305-1-AP), and TP53 (66304-1-Ig) primary antibodies. The membrane was subsequently incubated with secondary antibodies for 1 h at room temperature. Protein expression was detected and analyzed using the Bio-Rad ChemiDoc MP system.

### 2.8 Establishment and administration of animal models

#### 2.8.1 Establishment of animal models

Male BALB/c nude mice (4–6 weeks old, approximately 20 g each) were purchased from the Guangxi Medical University Animal Experiment Center. All animal experiments were approved by the Institutional Animal Care and Use Committee of the First Affiliated Hospital of Guangxi Medical University, Ethics No. 2024-E353-01. The ethical review adhered to the national standards GB/T35892-2018, “Guidelines for the Treatment of Laboratory Animals,” and “Guidelines for the Ethical Review of Laboratory Animal Welfare,” issued by the Ministry of Science and Technology of the People’s Republic of China. Hepa1-6 cells were cultured to the logarithmic growth phase, then digested and collected. BALB/c nude mice were subcutaneously injected with 0.1 mL of a suspension containing 1 × 10^6^ Hepa1-6 cells to establish a subcutaneous HCC xenograft model. Tumor growth was measured every other day.

#### 2.8.2 Administration

When tumors reached 100 mm^3^, the mice were randomly divided into four groups: NC group, RRx-001-L group (RRx-001, 5 μg/g), RRx-001-M group (RRx-001, 10 μg/g), and RRx-001-H group (RRx-001, 20 μg/g) (Das et al., 2016). Each group consisted of four mice. Intraperitoneal injections were given every 2 days, and the mice were observed for 7 days after three intraperitoneal injections to monitor mouse body weight and tumor growth. Tumor volume was calculated as (width (Sung et al., 2021) × length)/2. The experiment was terminated after 3 weeks of observation or before the tumor reached “20 mm × 20 mm”. Isoflurane anesthesia was used to kill mice and take tumors. Immunohistochemistry was performed to detect the expression of CD68 in the tumors of mice in the NC group and RRx-001-m group, and HE staining was performed on the heart, liver, spleen, kidney and other important organs of mice in the NC group and RRx-001-m group.

### 2.9 Statistical analysis

Experimental data were processed using Excel. SPSS 26.0 and ImageJ were employed for data analysis, and GraphPad Prism 8.0 was applied for image plotting. Quantitative data were expressed as mean ± standard deviation (M ± SD). Comparisons between two independent samples were made using t-test, and those among multiple groups were conducted using one-way ANOVA. All experiments were repeated three times. A *P*-value of <0.05 was considered statistically significant.

## 3 Results

### 3.1 Effect of RRx-001 on HCC cell proliferation

**FIGURE 1:**
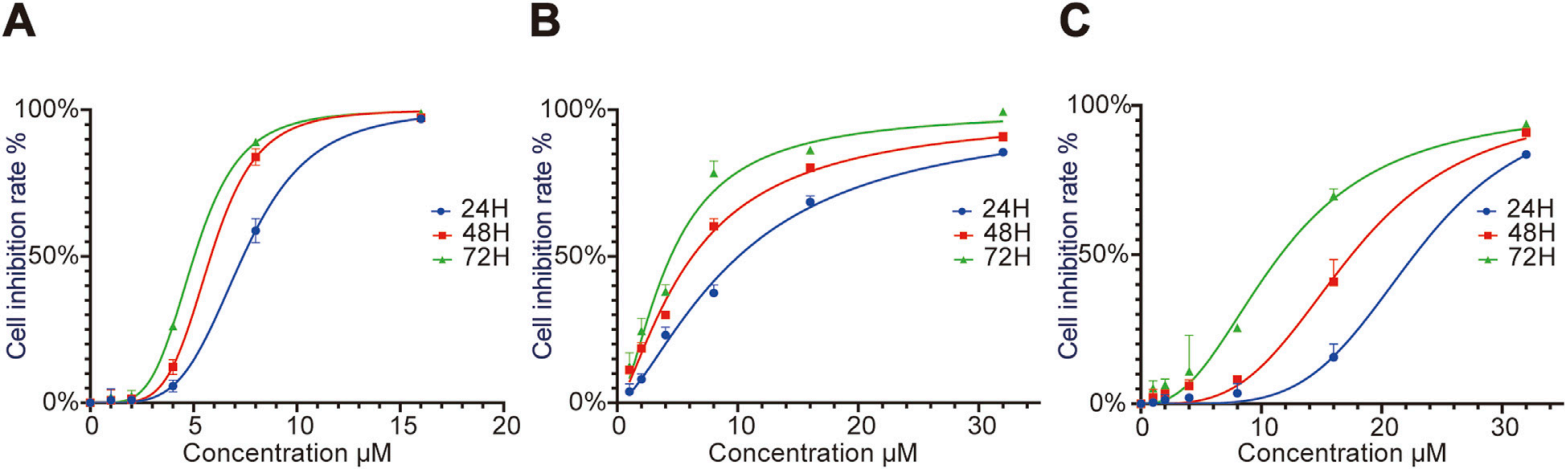
Effect of different RRx-001 concentrations on the proliferation of Huh7, Hepa1-6, andMHCC97H cells. Note: Huh7 **(A)**, Hepa1-6 **(B)**, and MHCC97H **(C)** cells were treated with RRx-001 for 24, 48, and 72 h, and cell proliferation inhibition rates were measured using the CCK8 kit.

CCK8 assay results showed that the IC_50_ values of RRx-001 against Huh7 cells at 24, 48, and 72 h were 7.55, 5.67, and 5.33 μmol/L, respectively. The IC_50_ values for Hepa1-6 cells at 24, 48, and 72 h were 11.53, 8.03, and 5.09 μmol/L, respectively; and those for MHCC97H cells were 20.72, 18.38, and 16.02 μmol/L, respectively. Cell proliferation assays demonstrated that RRx-001 significantly inhibited the proliferation of Huh7, Hepa1-6, and MHCC97H cells in a concentration- and time-dependent manner. The following experimental groups were subsequently established based on the IC_50_ values at 24 h for Huh7, Hepa1-6, and MHCC97H cells: control group, 0.5 × IC_50_ group, 1.0 × IC_50_ group, and 2.0 × IC_50_ group (Figures 1A–C).

### 3.2 Scratch assay to validate the effect of RRx-001 on HCC cell migration

**FIGURE 2:**
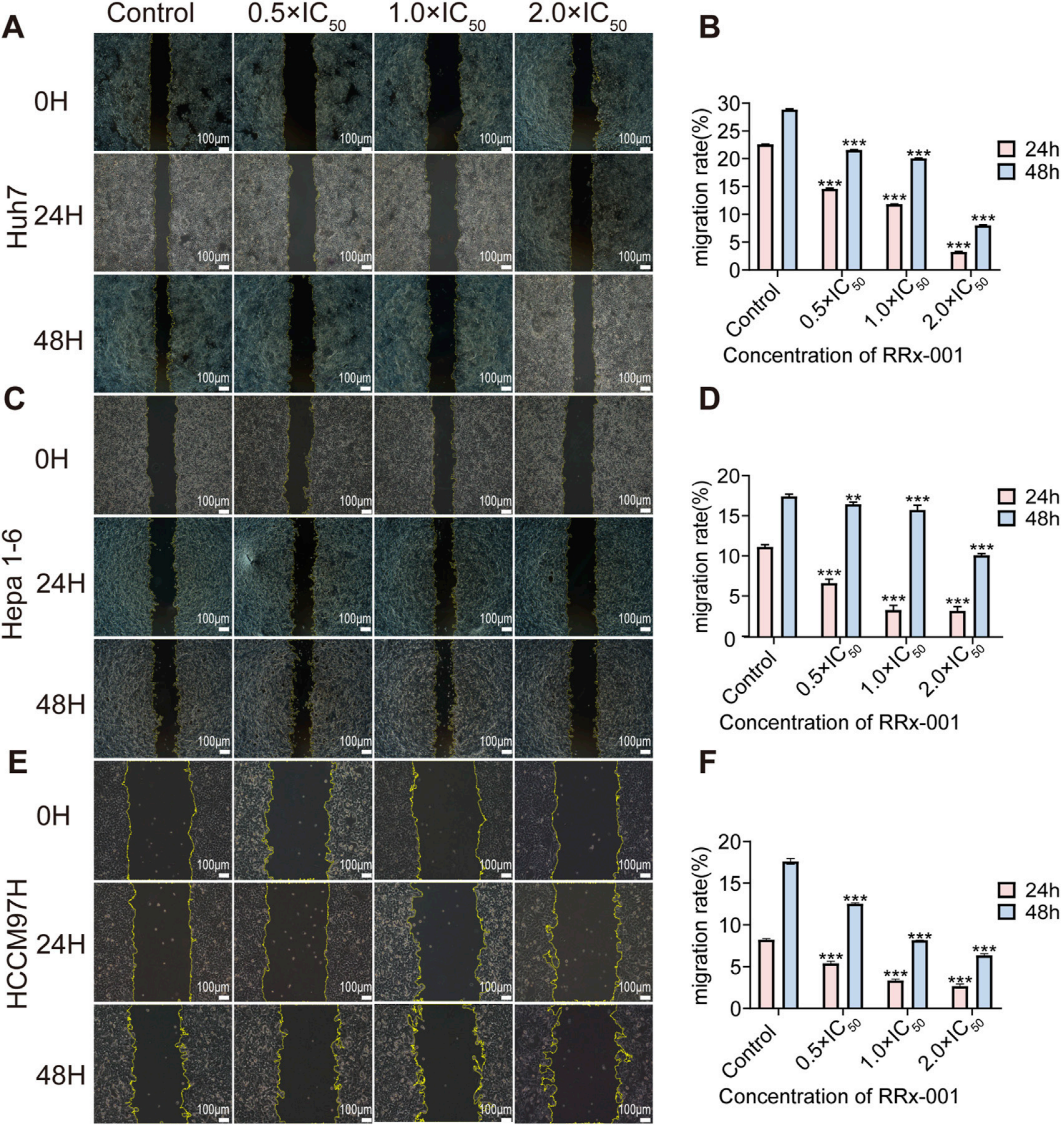
Impact of different RRx-001 concentrations on the scratch migration ability of Huh7, Hepa1-6, and MHCC97H cells. Note: Assessing the scratch healing and migration rates of Huh7 **(A, B)**, Hepa1-6 **(C, D)**, and MHCC97H **(E, F)** cells treated with RRx-001 for 24 and 48 h using scratch assay. Data are presented as the mean ± standard deviation of three experiments. Compared with the control group, *P < 0.05, **P < 0.01, ***P < 0.001. The corresponding concentrations for the control, 0.5 × IC_50_, 1.0 × IC_50_, and 2.0 × IC_50_ groups are as follows: Huh7 cells (3.5, 7, and 14 μM), Hepa1-6 cells (5.5, 11, and 22 μM), and MHCC97H cells (10, 20, and 40 μM).

With the increasing RRx-001 concentrations, the cell migration rate gradually decreased. The experimental results indicated that RRx-001 significantly inhibits the migration ability of Huh7 (Figures 2A, B), Hepa1-6 (Figures 2C, D), and MHCC97H cells (Figures 2E, F).

### 3.3 Transwell migration assay to validate the impact of RRx-001 on HCC cell migration

**FIGURE 3:**
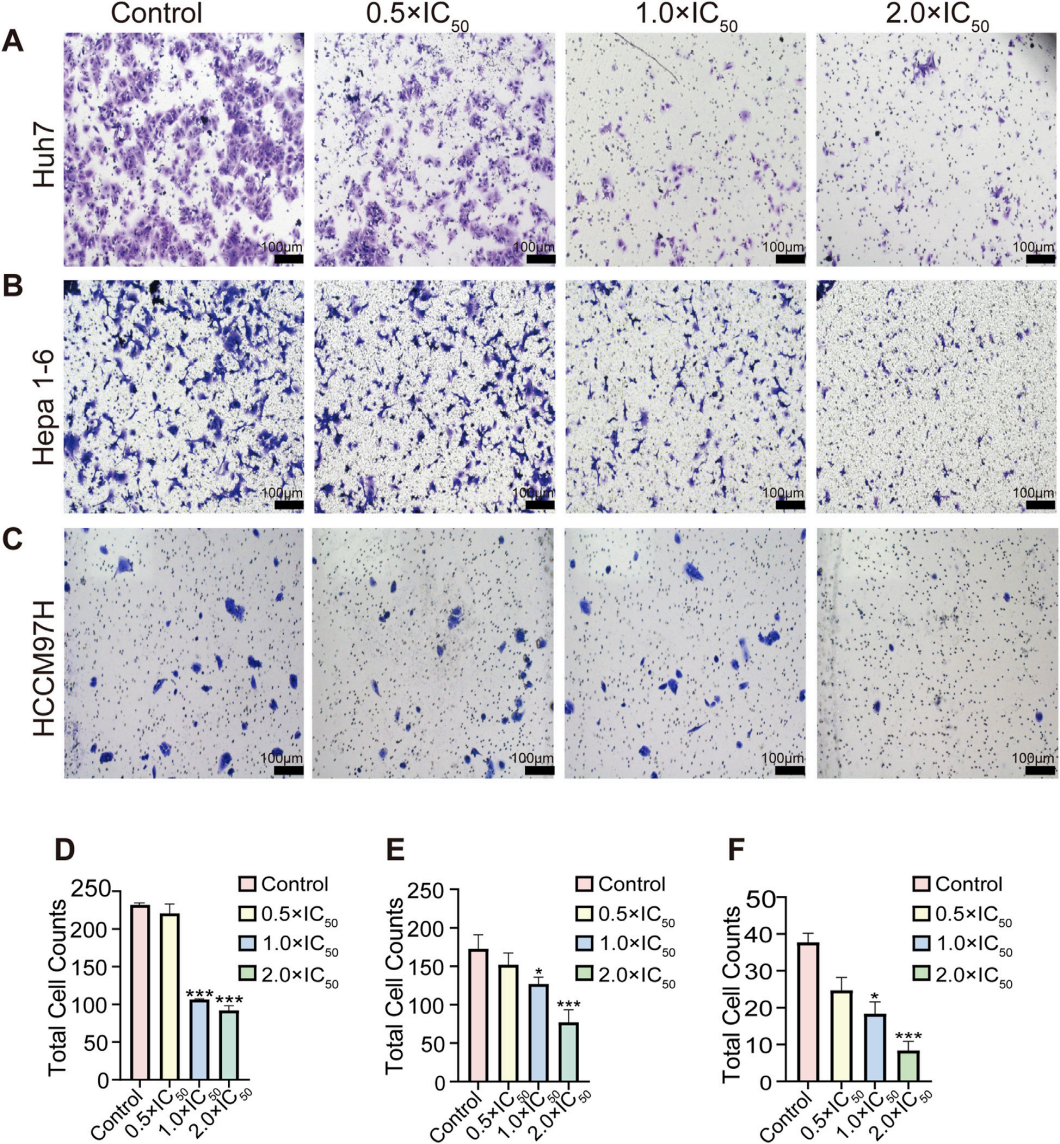
Impact of different RRx-001 concentrations on the Transwell migration ability of Huh7, Hepa1-6, and MHCC97H cells. Note: The influence of varying RRx-001 concentrations on the migration ability of Huh7 **(A, D)**, Hepa1-6 **(B, E)**, and MHCC97H **(C, F)** cells was evaluated using the Transwell assay. Data are presented as the mean ± standard deviation of three independent experiments. Compared with the control group, *P < 0.05, **P < 0.01, ***P < 0.001. Concentrations corresponding to the control, 0.5 × IC_50_, 1.0 × IC_50_, and 2.0 × IC_50_ groups were as follows: Huh7 cells (3.5, 7, and 14 μM), Hepa1-6 cells (5.5, 11, and 22 μM), and MHCC97H cells (10, 20, and 40 μM).

Transwell migration assay results indicated that the increasing RRx-001 concentrations lead to a reduction in cell migration rates. This finding demonstrated RRx-001s capability to inhibit the migration of Huh7 (Figures 3A, D), Hepa1-6 (Figures 3B, E), and MHCC97H (Figures 3C, F) cells.

### 3.4 Influence of RRx-001 on HCC cell invasion

**FIGURE 4:**
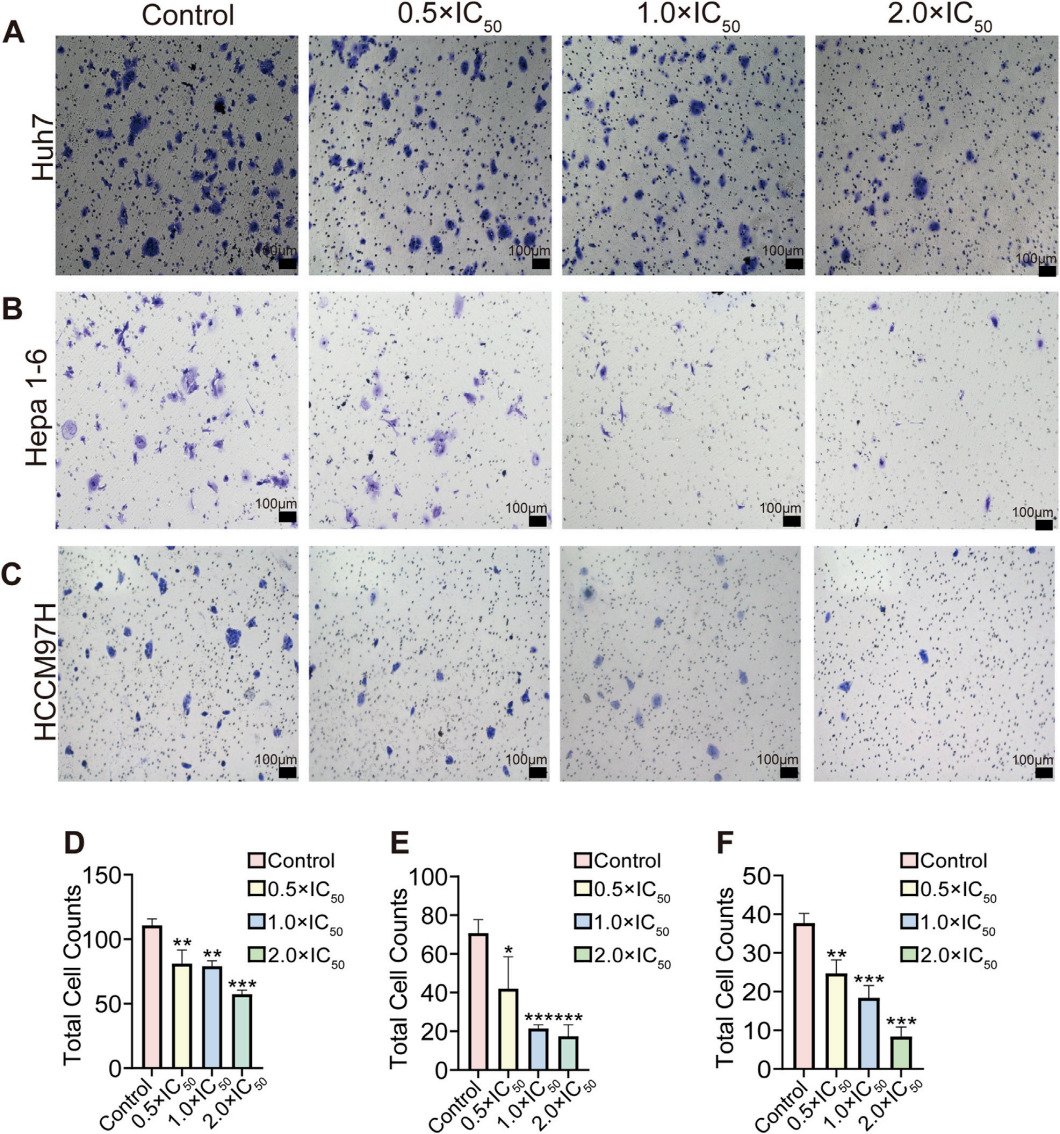
Effects of different concentrations and total number of RRx-001 treatment on the invasion capacity of Huh7, Hepa1-6, and MHCC97H cells. Note: The number of invasive Huh7 **(A, D)** Hepa1-6 **(B, E)**, and MHCC97H **(C, F)** cells treated with RRx-001 for 48 h was assessed using the Transwell assay. Data are presented as the mean ± standard deviation of three experiments. Compared with the control group, *P < 0.05, **P < 0.01, ***P < 0.001. The corresponding concentrations for the control, 0.5 × IC_50_, 1.0 × IC_50_, and 2.0 × IC_50_ groups are as follows: Huh7 cells (3.5, 7, and 14 μM), Hepa1-6 cells (5.5, 11, and 22 μM), and MHCC97H cells (10, 20, and 40 μM).

The Transwell invasion assay results showed a significant reduction in the number of invading cells in the RRx-001 group. In particular, the number of invading cells gradually decreased with the increasing RRx-001 concentration. These results suggested that RRx-001 can weaken the invasion capability of Huh7 (Figures 4A, D), Hepa1-6 (Figures 4B, E), and MHCC97H (Figures 4C, F) cells.

### 3.5 Effect of RRx-001 on HCC cell apoptosis

**FIGURE 5:**
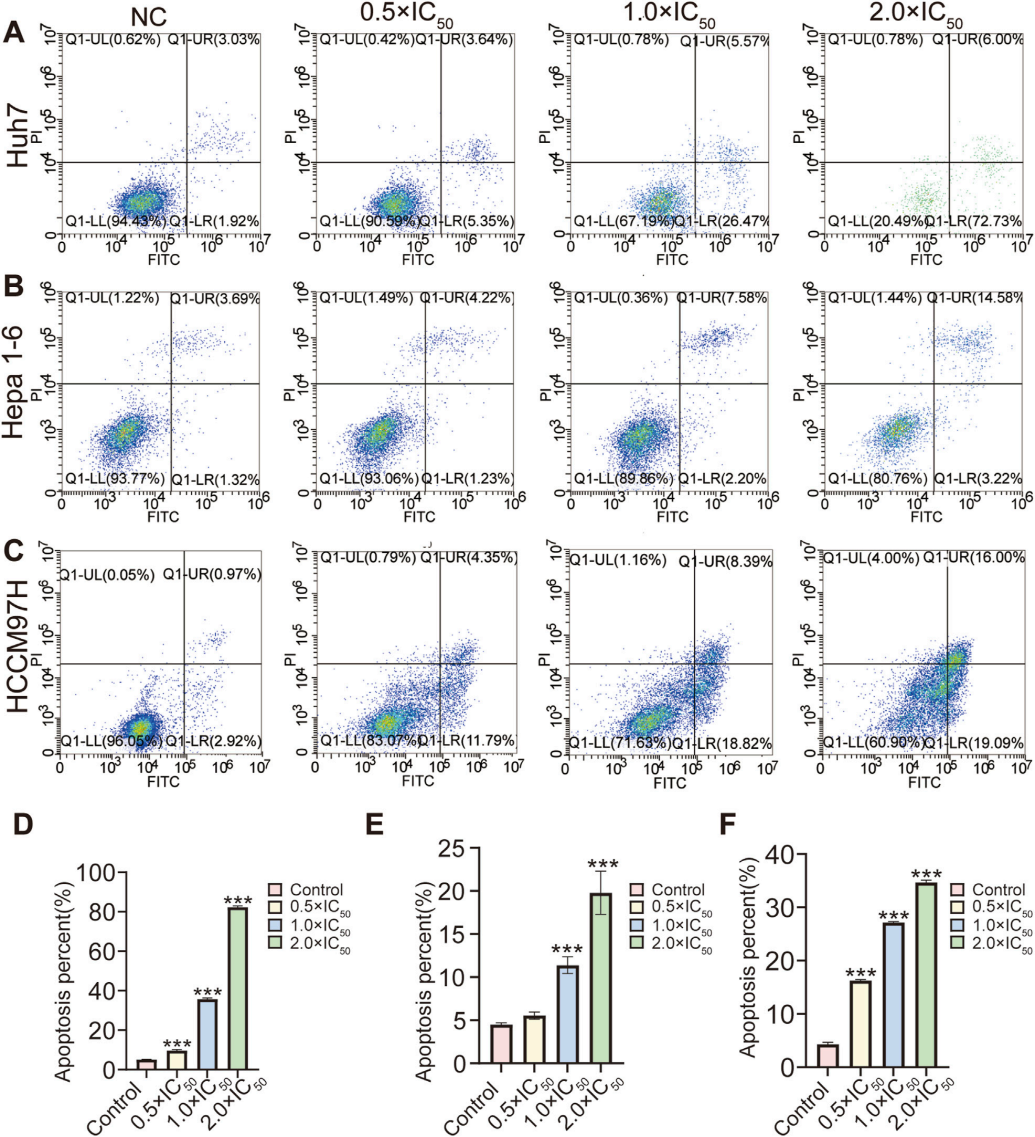
Effects of various RRx-001 concentrations on the apoptosis distribution and rates of Huh7, Hepa1-6, and MHCC97H cells. Note: Huh7 **(A, D)** Hepa1-6 **(B, E)**, and MHCC97H **(C, F)** cells were treated with various RRx-001 concentrations for 24 h. Apoptosis rates were assessed using flow cytometry. Data are presented as the mean ± standard deviation of three experiments. Compared with the control group, *P < 0.05, **P < 0.01, ***P < 0.001. The corresponding concentrations for the control, 0.5×IC_50_, 1.0 × IC_50_, and 2.0 × IC_50_ groups are as follows: Huh7 cells (3.5, 7, and 14 μM), Hepa1-6 cells (5.5, 11, and 22 μM), and MHCC97H cells (10, 20, and 40 μM).

HCC cells (Huh7, Hepa1-6, and MHCC97H) treated with different RRx-001 concentrations were analyzed for apoptosis using flow cytometry. The experimental results showed an increase in the apoptosis rates of (Figures 5A, D), Hepa1-6 (Figures 5B, E), and MHCC97H (Figures 5C, F) cells with the increasing RRx-001 concentrations, indicating that RRx-001 can significantly induce apoptosis in HCC cells.

### 3.6 Mechanistic study of RRx-001 in HCC

**FIGURE 6:**
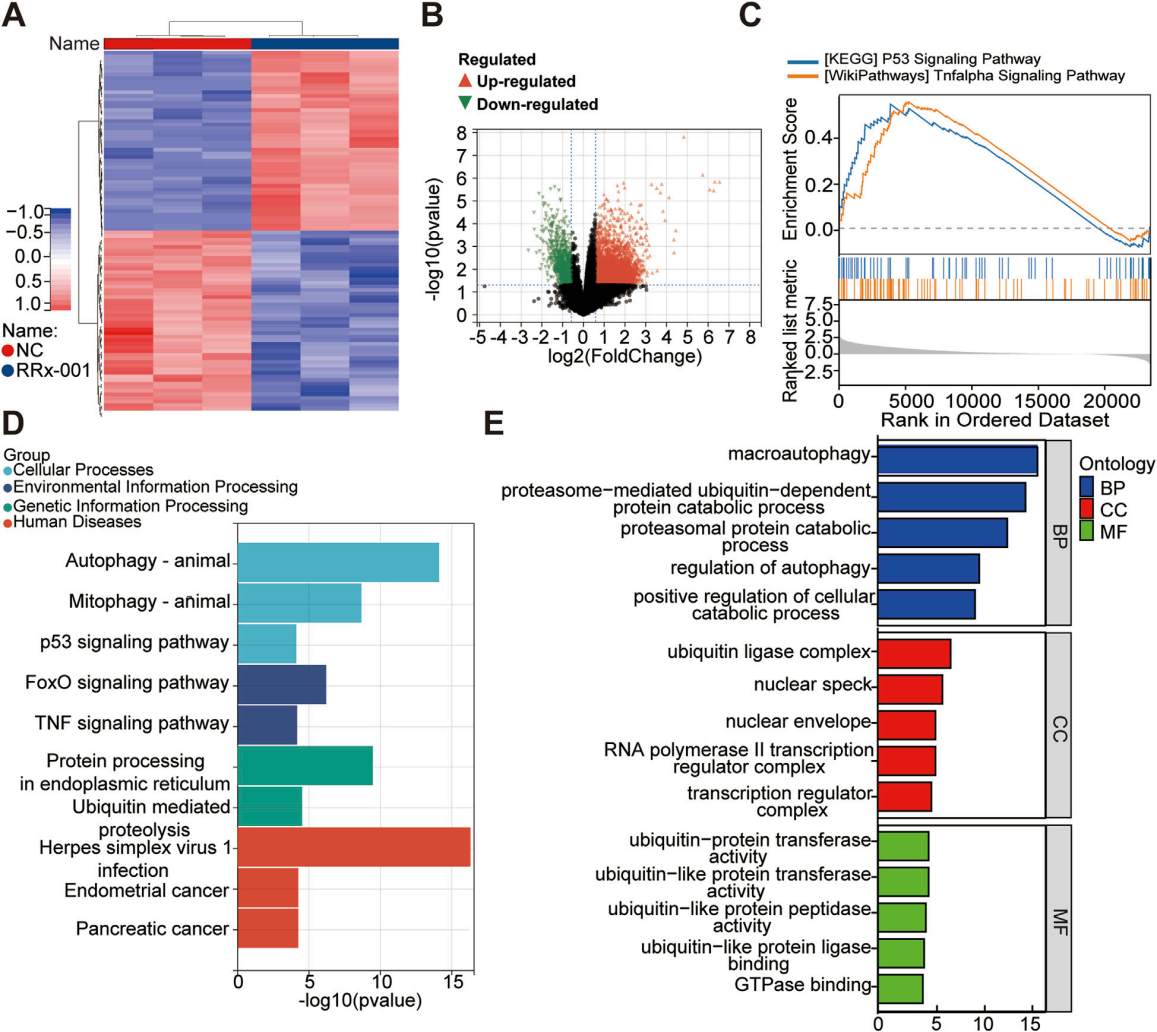
Whole transcriptome sequencing data for Huh7 cells treated with RRx-001. Note: Hierarchical clustering heatmap of differentially expressed genes **(A)**. Volcano plot showing expression differences **(B)**. Enrichment analysis plots from GSEA **(C)**. Bar chart of KEGG pathway analysis **(D)**. Bar chart of significantly differentially expressed mRNA genes enriched in GO terms **(E)**.

Huh7 cells were treated with RRx-001 for 24 h, and untreated Huh7 cells grown under normal conditions served as the blank control group. Whole transcriptome sequencing was conducted, followed by differential analysis and enrichment analyses including KEGG, GO, and GSEA. Differential expression analysis revealed 4,765 mRNA transcripts (4,044 upregulated and 721 downregulated) with significant differences between the treatment and control groups (Figures 6A, B). Clustering analysis of differentially expressed mRNA profiles was performed to identify distinct gene expression patterns. GO enrichment analysis of differentially expressed mRNAs highlighted their involvement in metabolic processes, cellular components, and cellular processes (Figure 6E). KEGG pathway enrichment analysis of genes significantly affected by RRx-001 indicated potential impacts on pathways such as P53 signaling, MAPK signaling, RAS signaling, TNF signaling, endocytosis, and RNA transport (Figure 6D). GSEA enrichment analysis of relevant differential genes suggested associations with P53 and TNF signaling pathways (Figure 6C). GO is enriched to GTPase binding, and Rho GTPases are involved in regulating cytoskeleton remodeling, cell migration, cell adhesion, cell polarization, gene transcription and other processes. This may be one of the mechanisms by which RRx-001 can inhibit liver cancer cell migration.

### 3.7 Analysis of Western blot results

**FIGURE 7:**
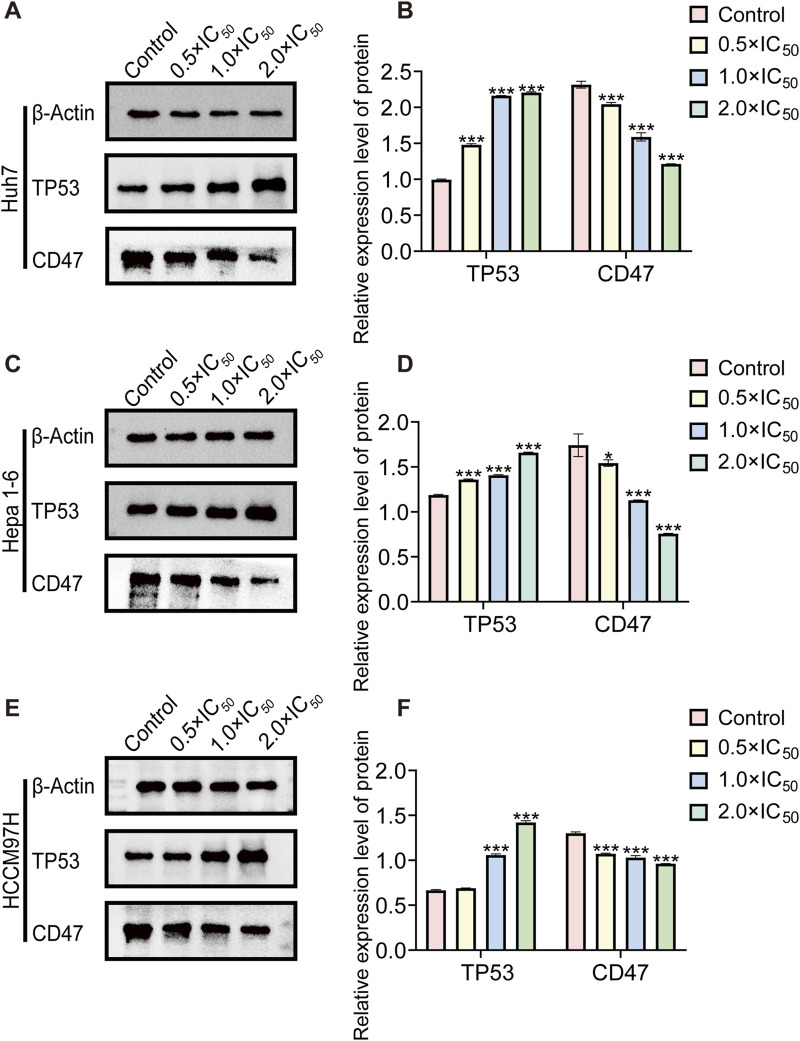
Western blot protein band diagram. Note: WB images and relative protein expression levels of TP53 and CD47in Huh7 **(A, B)** Hepa1-6 **(C, D)**, and MHCC97H **(E, F)** cells. Data are presented as the mean ± standard deviation of three experiments. Compared with the control group, *P < 0.05, **P < 0.01, ***P < 0.001.

As a CD47 inhibitor, RRx-001 demonstrates its cytotoxic effect on tumors by suppressing CD47 expression. KEGG enrichment analysis of sequencing data revealed that RRx-001s cytotoxic mechanisms against liver cancer cells are associated with the TP53 signaling pathway. Therefore, different RRx-001 concentrations were used to intervene in Huh7, Hepa1-6, and MHCC97H cells to verify RRx-001s mode of action in liver cancer. CD47 and TP53 expression levels were assessed by Western blot. Compared with that in the control group, a significant increase in TP53 expression and a decrease in CD47 expression were observed for all HCC cells at 0.5 × IC_50_, 1.0 × IC_50_, and 2.0 × IC_50_ concentrations, reaching statistical significance (Figures 7A–F). On the basis of these experimental findings, that RRx-001 may suppress liver cancer cells by reducing CD47 expression and increasing TP53 levels.

### 3.8 Inhibition of HCC tumor growth by RRx-001

**FIGURE 8:**
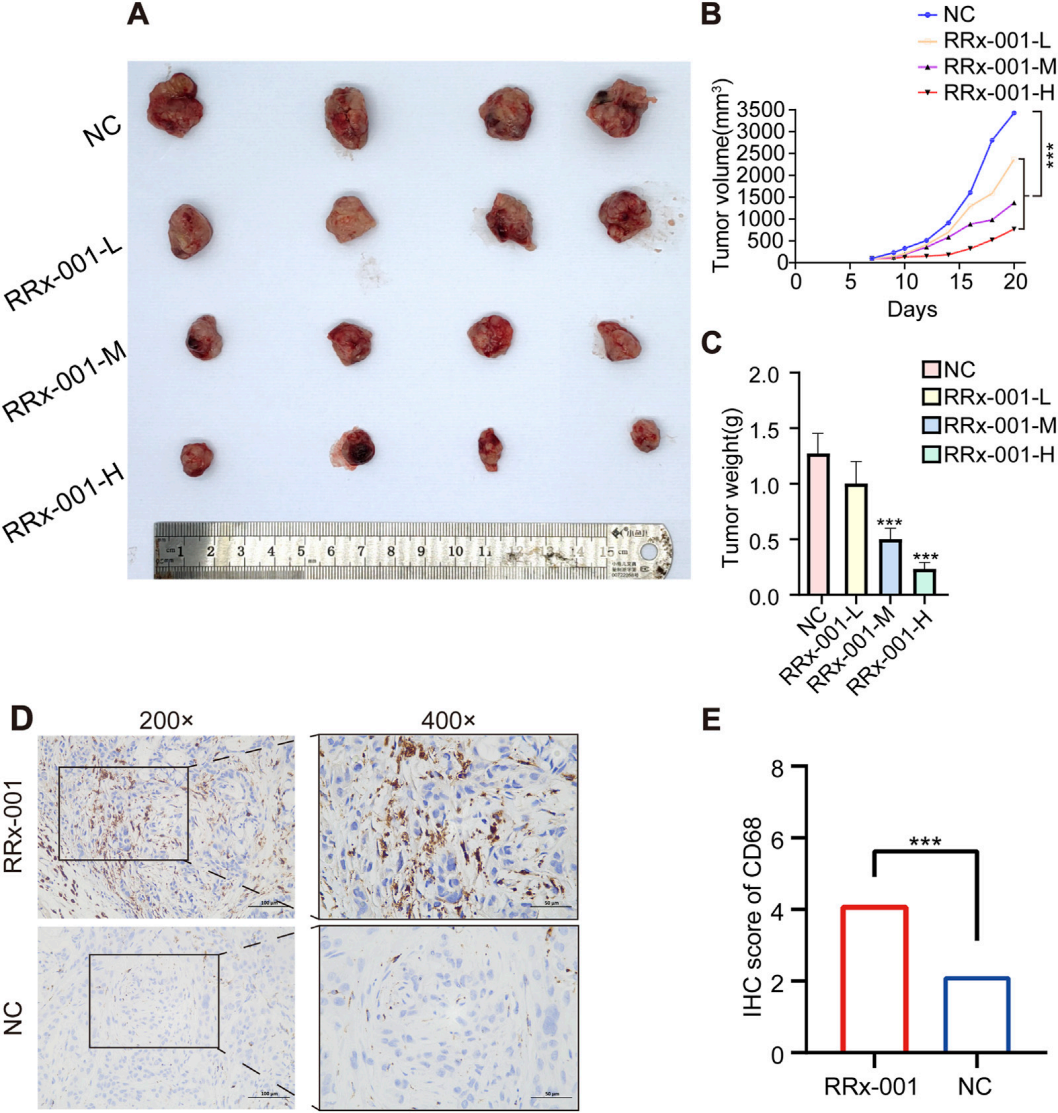
Effects of RRx-001 on HCC in nude mice. Note: Tumor images post dissection in each group **(A)**. Graph depicting the changes in tumor volume over time in each group **(B)**. Bar chart of tumor weight statistics **(C)**. Immunohistochemical results of CD68 in different groups **(D, E)**. Control group (NC), RRx-001-L group (5 μg/g), RRx-001-M group (10 μg/g), and RRx-001-H group (20 μg/g). Data are presented as mean ± standard deviation of four experiments, n = 4. (*P < 0.05, **P < 0.01, ***P < 0.001; compared to the model group).

There were no organic changes in the heart, liver, spleen, kidney and other important organs of mice in the RRx-001 group and the control group, indicating that the experimental dose of RRx-001 adopted this time did not produce therapeutic toxicity (Hematoxylin-eosin staining pictures are located in the Supplementary Material). The success rate of the xenograft mouse model of HCC was 100%. On the 7th day, the tumor nodules reached 100 mm^3^. After the corresponding treatment, the tumor volume of the control group increased significantly and the growth rate was fast, that in the RRx-001 group showed a slower increase, and that in the model group exhibited a significant increase (Figures 8A–C). The expression of CD68 in RRx-001 group was higher than that in control group (Figure 8D, E).

## 4 Discussion

HCC presents challenges due to the limitations of traditional therapies, making immunotherapy an increasingly prominent treatment option in recent years. The liver, as an immune organ and receptor of gastrointestinal antigens, creates a unique immune microenvironment. Key immune cells, such as Kupffer cells and cytotoxic T lymphocytes, play crucial roles in the development of HCC, providing ample opportunities for research into HCC immunotherapy (Jiang et al., 2023).Current studies suggest that RRx-001 mechanisms of anticancer activity involve downregulating CD47 to enhance chemotherapeutic immunity.

CD47 is a glycoprotein widely expressed in tumor cells and is highly expressed in patients with various cancers, including HCC, breast cancer, glioblastoma, lung cancer, colorectal cancer, and ovarian cancer. Its high expression is associated with low survival rates (Lo et al., 2015; Catalán et al., 2020; Upton et al., 2021; Jiang et al., 2022; Chen et al., 2023; Li et al., 2023; Hsieh et al., 2022). CD47 interacts with the SIRPα receptor on macrophages and dendritic cells, sending a “do not eat me” signal that protects healthy cells from being phagocytosed. Cancer cells exploit this mechanism by overexpressing CD47 on their surfaces, causing macrophages to recognize them as “normal” cells and thus evade macrophage-mediated phagocytosis. This overexpression allows tumor cells to avoid destruction by the immune system, facilitating immune evasion (Logtenberg et al., 2019; Jaiswal et al., 2009). Therefore, CD47 is an important target for immunotherapy, Downregulating CD47 expression can inhibit the SIRPα-CD47 axis, thereby enhancing innate and adaptive antitumor immune responses. RRx-001, a CD47 inhibitor, blocks the “do not eat me” signaling pathway by downregulating the CD47-SIRPα axis, thus preventing tumor cell immune evasion (Oronsky et al., 2020). Using Western blot analysis, we demonstrated that RRx-001 downregulates CD47 in Huh7, Hepa1-6, and MHCC97H cells. Consequently, RRx-001 may enhance antitumor immune responses by inhibiting the SIRPα-CD47 axis, restoring the phagocytic ability of macrophages against HCC cells and preventing immune evasion.

TAMs are the key immune cells in the Tumor microenvironment (TME). TAMs can be divided into M1 proinflammatory macrophages type and M2 anti-inflammatory macrophages type. Tumors can promote the increase of M2 macrophages by up-regulating the expression of CD47 to complete immune escape. Prevent phagocytosis by macrophages. It has been found that RRx-001 can lead to the increase of TAMs in tumors, and promote the transformation of M2 anti-inflammatory macrophages into M1 pro-inflammatory macrophages by down-regulating CD47 expression (Cabrales, 2019; Oronsky et al., 2017). The proliferation of M1 proinflammatory macrophages can produce immunogenic cytokines, reactive oxygen species (ROS) and active nitrogen (RNS) in hypoxic tumor, which can kill tumor cells (Yunna et al., 2020). CD68 is expressed in both M1 and M2 macrophages, but the expression level in M1 macrophages is higher than that in M2 macrophages, while the expression of CD68 in mouse tumors treated with RRx-001 is higher than that in NC group, which further confirms that RRx-001 can increase the number of TAMs by down-regulating the expression of CD47. It also promotes the transformation of M2 macrophages into M1 proinflammatory macrophages, thus playing an immunological role in tumor killing. In summary, RRx-001 requires TAMs to function, so TAMs are necessary for RRx-001 and can be a biomarker for the efficacy and prognosis of RRx-001.

These findings not only advance the progress of HCC immunotherapy based on existing research but also provide new perspectives for clinical studies in HCC treatment.

*P53* is one of the most common tumor suppressor genes, and its mutations are associated with various cancers. TP53 plays a critical role in maintaining genetic stability by regulating programmed cell death, cell cycle, DNA repair, and angiogenesis. Research has shown that *P53* mutations are closely linked to the development and progression of HCC. Mutant *P53* can acquire new functional activities, influencing numerous cellular processes and regulating the TME. For instance, mutant TP53 can promote tumor progression by altering exosome content, leading to the reprogramming of macrophages into the M2 phenotype, which creates a TME more conducive to liver cancer progression (Huang, 2021; Hernández Borrero and El-Deiry, 2021; Hu et al., 2021). RRx-001 exerts its anticancer effects by depleting cancer stem cells and activating the TP53 and Nrf2 pathways while providing some protection to normal cells (Oronsky et al., 2019b; Huang et al., 2022). Analysis of the transcriptome sequencing of RRx-001-intervened Huh7 HCC cell revealed the potential effects on the P53 signaling pathway. Western blot analysis also demonstrated that RRx-001 upregulates TP53 in Huh7, Hepa1-6, and MHCC97H cells. RRx-001 may exert its inhibitory effects on the proliferation, migration, and invasion of Huh7, Hepa1-6, and MHCC97H HCC cells by modulating the *P53*-related signaling cascade. Interestingly, it has been reported that RRx-001s sensitivity to tumor recovery is not affected by the status of P53, showing efficacy in both p53 mutations and wild-type tumors (Scicinski et al., 2015a). Combined with the experiment and previous studies, we speculated that one of the targets of RRx-001 in liver cancer is mutant P53, but whether it includes wild-type P53 needs further study.

Tumor migration refers to the process of tumor cells moving from the primary site to other tissues or organs, which is related to cytoskeletal recombination, TME, epithelial-mesenchymal transformation (EMT), immune escape and suppressation. The migration and metastasis of tumor cells is an important cause of poor prognosis in patients with liver cancer, so effectively inhibiting the migration of hcc has important clinical significance. Common drugs that inhibit hcc migration include Sorafenib, Regorafenib, and Cabozantinib, which work primarily by inhibiting tumor angiogenesis and interfering with tumor cell signaling pathways. However, unlike these drugs, RRx-001 inhibits hcc cell migration and metastasis by inhibiting CD47 signaling pathways, disrupting mitochondrial function, reducing ATP production and affecting cytoskeletal remodeling (Scicinski et al., 2015b; Brown and Frazier, 2001; Willingham et al., 2012; Tsai and Discher, 2008; Chao et al., 2011). This unique mechanism can not only effectively inhibit the invasion ability of tumor cells, but also enhance the body’s immune system to eliminate tumors, making RRx-001 has a unique advantage in the treatment of hcc migration. In addition, RRx-001 is less toxic to normal cells than other anti-migration agents and can better protect normal tissues in the treatment of hcc without significant systemic side effects.

HCC poses a global health threat, and many clinical anticancer drugs used for its treatment have significant adverse effects (Chen and Zhang, 2011; Gravitz, 2014; Li et al., 2021). Therefore, exploring an effective and low-toxicity novel anti-HCC drug is crucial. Derived from the aerospace industry, RRx-001 is a novel multi-effect anticancer drug with properties including macrophage activation, CD47 downregulation, vascular normalization, and epigenetic regulation, it exhibits potent anticancer effects against various cancers, It has been utilized in phase III registered clinical trials for third-line treatment and beyond in small cell lung cancer (Scicinski et al., 2015b; Zhang et al., 2021).RRx-001 may enhance the radiosensitivity of tumor cells by releasing NO, an endogenous vasodilator known to be an effective radiosensitizer. This release of NO can increase tumor blood flow, affect cellular respiration and signal transduction, and influence the production of reactive oxygen and nitrogen species (RONS), thereby making tumors more susceptible to radiation therapy (Scicinski et al., 2015b).Moreover, RRx-001 selectively delivers NO to tumor tissues with minimal systemic side effects on normal tissues, potentially significantly improving the therapeutic index of radiotherapy. As a low-toxicity anticancer drug, RRx-001 has been shown in several studies to have “minimal systemic side effects” on normal tissue, making it potentially useful in combination therapy. Studies have found that RRx-001 can work synergistically with checkpoint inhibitors (Criscuolo et al., 2019; Veillette and Chen, 2018), chemotherapy agents (Lee et al., 2021), targeted therapies (Brzezniak et al., 2016; Carter et al., 2016a; Carter et al., 2016b), anti-angiogenesis inhibitors (Reid et al., 2022; Scicinski et al., 2015a), radiotherapy (Oronsky et al., 2016) and other therapeutic approaches, all of which show the potential to enhance efficacy and reduce toxicity, making it an ideal combination therapy. With this combination treatment strategy, RRx-001 is expected to not only improve patient survival, but also improve the overall treatment experience.

Our transcriptomic analysis of RRx-001-intervened HCC cells revealed that RRx-001 may act against HCC through various signaling pathways, including the MAPK, RAS, TNF, and FoxO pathways. Therefore, RRx-001 is a therapeutic agent that exerts inhibitory effects on tumors by modulating multiple cancer-related signaling pathways and holds promising research potential for HCC treatment. This study provides an initial exploration of its mechanisms, suggesting that further investigations are warranted to fully elucidate its specific mechanisms.

## 5 Conclusion

RRx-001 effectively suppresses HCC cell viability by downregulating CD47 and upregulating TP53. This modulation of key signaling pathways inhibits the migration and invasion of HCC cells and induces apoptosis. *In vivo* studies have shown that RRx-001 inhibits tumor growth by increasing TAMs, indicating its potential as a therapeutic agent for HCC.

## Data Availability

The original contributions presented in the study are publicly available. This data can be found here: NCBI repository, accession number PRJNA1187898 (https://www.ncbi.nlm.nih.gov/bioproject/?term=PRJNA1187898/)
