# Supplementary figures and images for: Anti-tumor and anti-metastatic effects of RRx-001 on hepatocellular carcinoma: mechanisms of action and therapeutic potential
Source: Front Pharmacol. 2024 Nov 27;15:1469825. doi: 10.3389/fphar.2024.1469825 (PMC11631591; doi:10.3389/fphar.2024.1469825)

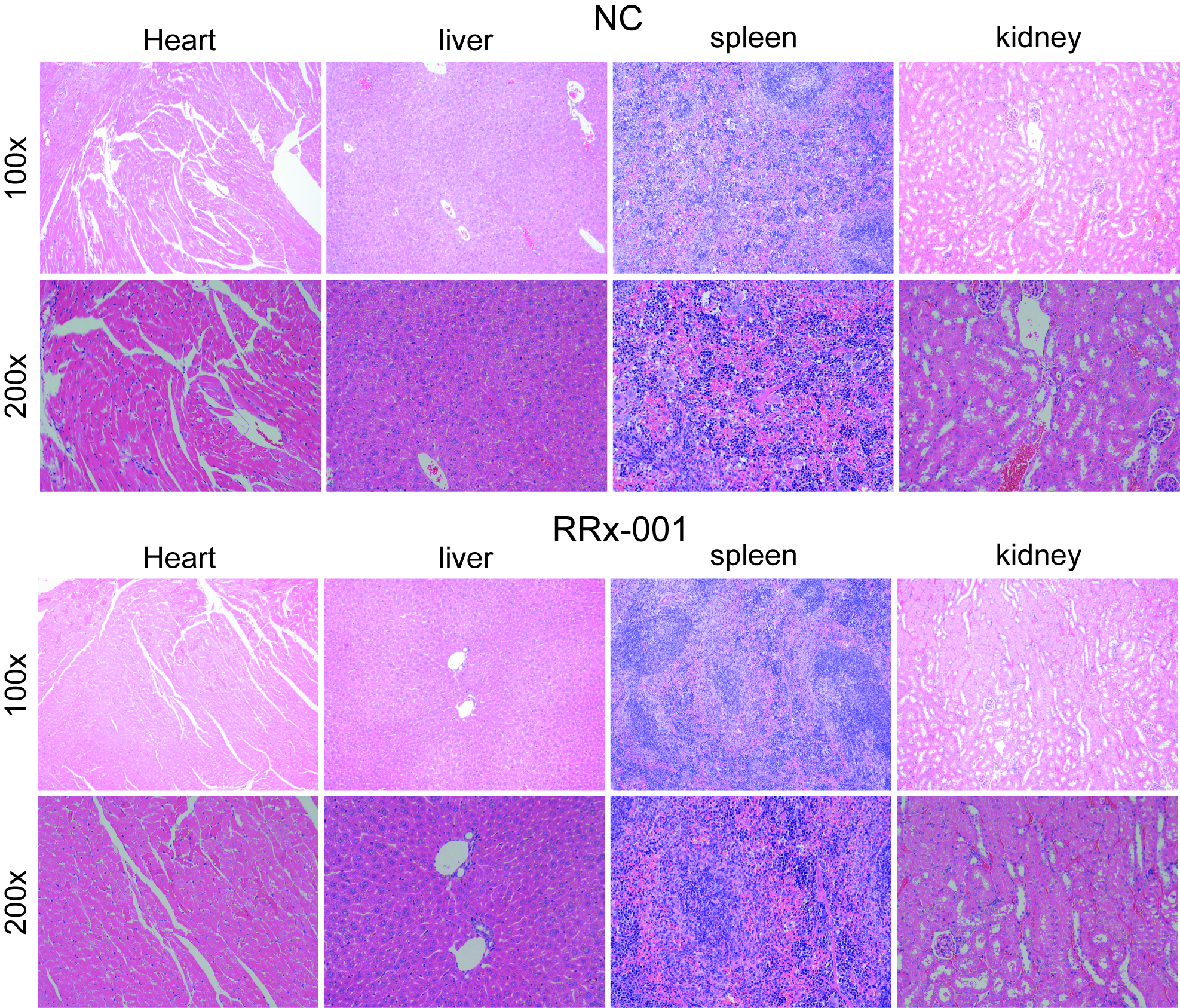

Supplement: Supplementary file 1 [file Image1.TIF]
